# Breastfeeding is associated with enhanced learning abilities in school-aged children

**DOI:** 10.1186/s13034-017-0169-0

**Published:** 2017-07-19

**Authors:** Johanna Inhyang Kim, Bung-Nyun Kim, Jae-Won Kim, Soon-Beom Hong, Min-Sup Shin, Hee Jeong Yoo, Soo-Churl Cho

**Affiliations:** 1 Department of Public Health Medical Services, Seoul National University Bundang Hospital, 173 Bun-gil 82, Goomi-ro, Bundang-gu, Seongnam, Gyeonggi-do 13620 Republic of Korea; 2 Division of Child and Adolescent Psychiatry, Department of Psychiatry, Seoul National University College of Medicine, 101 Daehak-no, Chongno-gu, Seoul, 03080 Republic of Korea; 3 Division of Child and Adolescent Psychiatry, Department of Psychiatry, Seoul National University Hospital, 101 Daehak-no, Chongno-gu, Seoul, 03080 Republic of Korea; 4 Department of Psychiatry, Seoul National University Bundang Hospital, 82, 173 Bun-gil, Gumi-ro, Bundang-gu, Seongnam, 13620 Republic of Korea; 5 Armed Forces Capital Hospital, 81 Bun-ji, 177 Bun-gil, Saemail-ro, Bundang-gu, Seongnam, Kyunggi-di 13573 Republic of Korea

**Keywords:** Breastfeeding, Intelligence, Learning, Maternal cognition

## Abstract

**Objective:**

The majority of studies on the associations between breastfeeding and cognitive functioning have focused on IQ, with only a few investigating learning skills, and none of the latter adjusting for maternal IQ. We examined the association between breastfeeding and learning abilities in school-aged children using a cross-sectional design.

**Methods:**

We recruited 868 children, aged 8–11 years and parents completed the Learning Disability Evaluation Scale (LDES). Multivariable linear regression models were used and age, gender, area of residence, annual family income, maternal education, and maternal age at delivery, were included as covariates. Maternal IQ was added to further adjust for the effects of maternal cognitive ability. Path analysis was conducted to investigate the mediation effect of maternal IQ between breastfeeding and learning skills.

**Results:**

Children who were ever-breastfed had higher learning quotient scores on the LDES (p = 0.001) as well as higher scores on subscales related to speaking (p = 0.001), reading (p = 0.005), writing (p = 0.004), spelling (p = 0.003), and mathematical calculation (p = 0.003) than the never-breastfed participants. All of these variables remained significant after adjusting for gestational and socioeconomic factors and for maternal IQ as covariates. The path analysis showed that breastfeeding had both indirect and direct effects on the learning quotient.

**Conclusions:**

The results suggest that breastfeeding is positively associated with learning skills in school-aged children.

## Background

Previous studies have reported that breastfeeding is linked to enhanced cognitive performance in childhood including superior attentional skills and a higher intelligence quotient (IQ) relative to children who were not breastfed [1]. A meta-analysis reported that breastfed children had an adjusted cognitive advantage of 3.15 IQ points compared to formula-fed children [2]. This positive effect of breastfeeding on IQ was also observed in a randomized trial, which reported an average 7.5 IQ points increase in children who were allocated to breastfeeding promotion groups [3]. However, given that breastfeeding mothers are more likely than non-breastfeeding counterparts to be older, have a higher socioeconomic status (SES), and engage in behaviors that stimulate child development [2, 4], some studies have reported that the association between breastfeeding and IQ is attenuated when SES variables and maternal IQ are controlled for [5]. For example, a meta-analysis reported that the 4-point increase of IQ in breastfed subjects relative to formula-fed subjects was attenuated to the point of non-significance after adjustment for maternal IQ [6]. This study concluded that breastfeeding has little or no effect on the intelligence of children [6]. Hence, it is important to consider the effect of maternal IQ in investigating the relationship between breastfeeding and children’s cognitive functioning.

Most of the studies investigating breastfeeding and cognitive abilities have focused on the child’s IQ, whereas only a few have investigated academic performance or learning skills, and these studies have produced inconsistent results. A previous study reported that, after adjustment for SES factors, children who were breastfed showed a significant test score advantage in reading and mathematics compared to those who were not [7]. Another study yielded negative results, reporting that the home learning environment was more important than predominant breastfeeding in determining early-age math and reading skills [8]. Moreover, some studies have assessed academic performance using teachers’ reports, whereas others have used standardized tests that measure learning skills in specific cultural contexts [7, 9]. None have used a standardized tool that can be widely applied to children of various cultural backgrounds. Furthermore, none of these studies have adjusted for the effects of maternal IQ; considering the highly predictive value of maternal IQ on children’s cognitive abilities, this may have confounded the results.

In the present study, we investigated the relationship between breastfeeding and learning abilities, using the Learning Disability Evaluation Scale (LDES), a widely used screening instrument to evaluate various learning skills, screen for learning disabilities, and calculate the learning quotient (LQ). We hypothesized that the association between breastfeeding and learning abilities would be significant, even after adjusting for various developmental and SES variables and for maternal IQ.

## Methods

### Participants

This study used a cross-sectional design and was conducted as a 3-year project whose detailed protocols have been described elsewhere [10, 11]. Third and fourth graders (aged 8–11 years) were recruited in five administrative regions in South Korea, including Seoul and Seongnam (urban districts), Incheon and Ulsan (industrial districts) and Yeoncheon (rural district). We selected the two or three schools in each region that were most representative of the local demographics, and letters of invitation were sent to the parents of the participants (n = 1712). Schools in the center of each region were chosen, to reflect a microcosm of each region. Detailed information was provided to all parents, and written informed consent was obtained prior to enrollment. The study protocol was approved by the institutional review board of the Seoul National University Hospital.

The participants’ parents filled out questionnaires that contained items inquiring about gestational, socioeconomic and developmental factors. Infant feeding methods were investigated using an item inquiring whether the child had ever been breastfed. Maternal IQ was measured using the short form of the Korean Wechsler Adult Intelligence Scale under the guidance of a trained examiner who was blind whether the children had been breastfed or not. Short forms are known to correlate well with full scale IQ [12].

### Measurement of learning abilities

Parents of the participants completed the LDES, a parent-rated scale consisting of 88 items [13]. Items yield scores on a scale of 1–3, and results are presented as age-adjusted scores on seven subscales pertaining to listening, thinking, speaking, reading, writing, spelling and mathematical calculation. Higher scores indicate better performance, and the subscores are summed to calculate an overall LQ. The Korean version of the LDES has been age-standardized and has been found to have fair reliability and validity [14].

### Statistical analyses

The demographic and clinical characteristics of the ever-breastfed and never-breastfed participants, as well as the participants who were excluded from the analysis, were compared using independent t tests for continuous variables and Chi square tests for categorical variables.

We analyzed the association between LDES subscale scores and breastfeeding using multivariable linear regression models. Graphic and residual analyses were performed to assess modelling assumptions. Univariable regression was used to investigate the association between breastfeeding and demographic and clinical factors (age [years], gender [male or female], area of residence [urban, industrial or rural], annual family income [$25,000 and higher or below $25,000], maternal education level [years], birth weight [kg], gestational age at birth [weeks], age of mother at birth [years], and maternal IQ). We included those variables found to be statistically significant (p < 0.10) as covariates. None of the multivariable linear regression models revealed multicollinearity among the independent variables. A path analysis was conducted to investigate the direct effect and also the indirect effect mediated by maternal IQ of breastfeeding on LQ scores.

All statistical analyses were performed using SPSS (version 22.0; SPSS Inc., Chicago, IL, USA) and AMOS (version 18.0; SPSS Inc., Chicago, IL, USA). Statistical significance was defined as a p value <0.05.

## Results

Among the initial 1712 participants we solicited, 1089 (response rate 63.6%) agreed to participate in the study, and data related to breastfeeding and the LDES was obtained for 868 (79.7%; 453 boys and 415 girls, mean age 9.0 ± 0.7) children. Among these 868 participants, 516 (59.4%) were ever-breastfed during infancy and 352 (40.6%) were never-breastfed.

The demographic and clinical characteristics of the ever-breastfed and never-breastfed participants included in the study, as well as the 221 who were excluded from the analyses, are presented in Table 1. Maternal IQ and birth weight were higher in the ever-breastfed group compared to the never-breastfed group. There were no differences between the two groups with regard to other demographic and SES characteristics.

**Table 1:** Demographic and clinical characteristics of the breastfed and non-breastfed participants, and of participants excluded from analyses

Characteristics	Ever-breastfed participants (n = 516)	Never-breastfed participants (n = 352)	p value	Excluded participants (n = 221)	p value
Age (years), mean (SD)	9.1 (0.7)	9.0 (0.7)	0.337	9.1 (0.7)	0.041
Sex (male), N (%)	267 (51.7)	186 (52.8)	0.751	118 (53.4)	0.749
Annual family income >$2500, N (%)	311 (60.9)	229 (65.4)	0.173	81 (59.6)	0.480
Paternal education, years, mean (SD)	13.8 (2.2)	13.8 (2.2)	0.928	13.2 (2.3)	0.001
Maternal education	13.3 (2.0)	13.1 (2.1)	0.118	12.8 (2.2)	0.043
Region			0.098		<0.001
Urban	220 (42.6)	128 (36.4)		115 (52.0)	
Industrial	214 (41.5)	152 (43.2)		56 (25.3)	
Rural	82 (15.9)	72 (20.5)		50 (22.6)	
Birth weight	3.3 (0.5)	3.2 (0.5)	<0.001	3.2 (0.5)	0.886
Maternal age at birth	28.3 (3.8)	28.4 (3.8)	0.875	28.9 (4.9)	0.245
Maternal IQ	108.1 (11.4)	106.4 (11.9)	0.039	108.4 (10.1)	0.886
Gestational age at birth	39.8 (1.2)	39.7 (1.6)	0.241	39.8 (0.8)	0.213

*SD* standard deviation, *IQ* intelligence quotient

The association of potential covariates with breastfeeding is presented in Table 2. Among the significant factors (p < 0.10), age, gender, area of residence, annual family income, years of maternal education, and age of mother at birth were included as covariates in Model 2 and maternal IQ was additionally included in Model 3 to adjust for maternal cognition.

**Table 2:** Association of demographic and clinical characteristics with learning quotient

Characteristic	B (SE)	95% CI	p value
Age	−1.35 (0.48)	−2.30, −0.40	0.005
Gender	3.58 (0.68)	2.25, 4.91	<0.001
Area of residence			
Urban	R	R	R
Industrial	−0.54 (0.76)	−2.03, 0.94	0.473
Rural	−1.80 (0.98)	−3.71, 0.12	0.067
Annual family income	−3.17 (0.71)	−4.55, −1.78	<0.001
Years of maternal education	0.64 (0.16)	0.33, 0.96	<0.001
Birth weight	0.41 (0.74)	−1.03, 1.86	0.574
Gestational age at birth	−0.40 (0.36)	−0.91, 0.11	0.122
Age of mother at birth	−0.16 (0.09)	−0.34, 0.02	0.082
Maternal IQ	0.13 (0.03)	0.07, 0.19	<0.001

The association between LDES subscale scores and breastfeeding is presented in Table 3. In Model 1, the ever-breastfed participants had higher overall LQ scores (p = 0.001) as well as higher scores on subscales related to speaking (p = 0.001), reading (p = 0.005) writing (p = 0.004), spelling (p = 0.003) and mathematical calculation (p = 0.003). After adjusting for age, gender, area of residence, annual family income, years of maternal education, birth weight, gestational age at birth, and maternal age at birth, the same variables as those in Model 1 emerged as significant, with ever-breastfed children having higher LQ scores (p = 0.004) than never-breastfed children as well as having higher scores on subscales related to speaking (p = 0.001), reading (p = 0.011), writing (p = 0.012), spelling (p = 0.006), and mathematical calculation (p = 0.028). When maternal IQ was added as a covariate in Model 3, all of these variables remained significant, i.e., LQ scores (p = 0.005) as well as subscores for speaking (p = 0.001), reading (p = 0.015), writing (p = 0.022), spelling (p = 0.013), and mathematical calculation (p = 0.026). The effect sizes (Cohen’s f^2^) for each subscale in Model 3 ranged from 0.041 to 0.117, indicating small effect sizes.

**Table 3:** Association of LDES scores with breastfeeding

LDES score, mean (SD)	Ever-breastfed participants (N = 516)	Never-breastfed participants (N = 352)	Model 1		Model 2^a^		Model 3^b^		Effect size (Model 3)
			Β (95% CI)	p value	Β (95% CI)	p value	Β (95% CI)	p value	
LDES									
Listening	11.4 (2.2)	11.2 (2.5)	0.28 (−0.04, 0.60)	0.085	0.35 (−0.71, 0.57)	0.126	0.30 (−0.04, 0.63)	0.080	0.056
Thinking	11.4 (1.8)	11.2 (2.1)	0.23 (−0.04, 0.50)	0.089	0.19 (−0.70, 0.50)	0.150	0.19 (−0.08, 0.46)	0.165	0.041
Speaking	11.7 (2.0)	11.2 (2.3)	0.49 (0.21, 0.78)	0.001	0.50 (0.21, 0.79)	0.001	0.49 (0.20, 0.79)	0.001	0.058
Reading	11.3 (2.1)	10.8 (2.4)	0.44 (0.14, 0.74)	0.005	0.38 (0.67, 0.99)	0.011	0.37 (0.07, 0.67)	0.015	0.076
Writing	11.3 (2.0)	10.9 (2.4)	0.43 (0.14, 0.73)	0.004	0.47 (0.08, 0.65)	0.012	0.34 (0.05, 0.63)	0.022	0.101
Spelling	10.9 (2.3)	10.4 (2.8)	0.52 (0.18, 0.86)	0.003	0.46 (0.13, 0.78)	0.006	0.42 (0.09, 0.76)	0.013	0.117
Mathematical calculation	11.8 (1.5)	11.4 (2.1)	0.37 (0.12, 0.61)	0.003	0.26 (0.03, 0.50)	0.028	0.27 (0.03, 0.51)	0.026	0.048
Learning quotient	108.8 (9.0)	105.6 (11.4)	2.23 (0.86, 3.59)	0.001	1.97 (0.65, 3.30)	0.004	1.95 (0.59, 3.31)	0.005	0.092

*LDES* Learning disability evaluation scale, *SD* standard deviation

^a^ Covariates include age, gender, area of residence, annual family income, years of maternal education, and age of mother at birth

^b^ Covariates include age, gender, area of residence, annual family income, years of maternal education, age of mother at birth, and maternal IQ

**Fig. 1:**
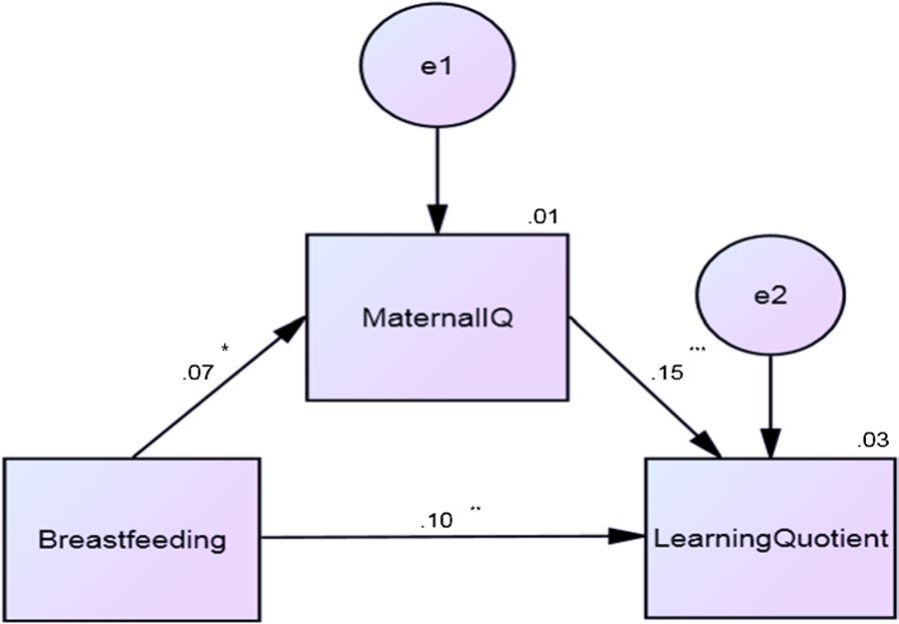
Path model of breastfeeding and the learning quotient, mediated by maternal IQ. *IQ* intelligence quotient. *p < 0.05, **p < 0.01, ***p < 0.001

In the path analysis, the degree of freedom was 0, indicating a saturated model. The results of the path model were shown in Fig. 1. Breastfeeding had both direct and indirect effect on the LQ. Breastfeeding was significantly associated with maternal IQ (β = 0.072, p = 0.039), and maternal IQ was significantly associated with LQ scores (β = 0.145, p < 0.001). The direct effect between breastfeeding and LQ was 0.098 (p = 0.003), and the indirect effect was 0.010. The squared multiple correlation was 0.03 in the LQ, indicating that this model accounted for 3% of variance of the LQ.

## Discussion

This study found that those who were ever-breastfeed had a positive advantage on learning skills including speaking, reading, writing, spelling and mathematical calculation compared to those that were never-breastfed, even after controlling for various gestational and socioeconomic variables. This is the first study to examine the association between breastfeeding and learning skills with inclusion of maternal IQ as a covariate. The path-analysis revealed that breastfeeding had both a direct and indirect effect on learning skills, and the indirect effect was mediated by maternal IQ. This is meaningful given that several studies have found the association between breastfeeding and children’s cognitive abilities to be non-significant after adjustment for maternal intelligence [6]. One strength of the current study is that we found a significant association between ever-breastfeeding and a variety of skills related to speaking, reading, writing, spelling, and mathematical calculation as well as with the overall LQ.

The results of this study are in line with those of previous studies that reported a positive association between breastfeeding and academic achievement. An 18-year longitudinal study reported higher reading and mathematical skills between 10 and 13 years of age among breastfed children relative to formula-fed children [15], and the Western Australian Pregnancy Cohort Study found that predominant breastfeeding was positively associated with mathematical and reading achievement at age 10 [9]. Both of these studies suggest, as does the present study, that the benefits of breastfeeding are found not only in early childhood, but persist into late childhood. Although much is not known on the long-term effect of breastfeeding, a recent study reported that breastfed subjects have higher educational attainment and higher income in adulthood [16].

The mechanism responsible for the protective effects of breastfeeding on cognitive functioning has been thought to be related to nutrients in breast milk that are essential for optimal brain growth. Long-chain polyunsaturated fatty acids, including docosahexaenoic (DHA) acid and arachidonic (AA) acid, are structural elements that are essential in the formation of new tissue, including neurons [17]. DHA and AA comprise approximately 20% of the fatty acid content of the brain and are involved in early neurodevelopment, promoting neuronal growth, repair and myelination [18]. Moreover, other studies have reported that breastfed and formula-fed infants exhibit different gut microbial profiles, a factor that is also related to brain and myelin development [19]. The effects of breastfeeding on brain development have been further supported by several magnetic resonance imaging studies. Morphometric studies conducted with adolescents have demonstrated that breastfed subjects exhibited increased total white matter, subcortical gray matter and parietal lobe cortical thickness, and have reported a relationship between duration of breastfeeding and IQ [20–22]. Another study of 133 healthy children between 10 months and 4 years of age reported that breastfed children had increased white matter integrity in the frontal and temporal white matter, the peripheral regions of the internal capsule and corticospinal tracts, and in the superior longitudinal fasciculus and superior occipital-frontal fasciculus [23]. These are areas related to higher-order cognition, a domain in which breastfed children have been found to exhibit enhanced performance.

The excluded participants were significantly older, the paternal education level was lower, and a higher proportion of participants lived in urban and rural areas, with a lower proportion living in industrial regions compared to those included in the analyses. As there was a significant negative association between age and the LQ, and also a significant positive relationship between paternal education level and the LQ, there is a possibility that participants with poor learning abilities were more likely to have been in excluded from the analyses rather than being included. Therefore, cautious interpretation of this data is required.

The World Health Organization (WHO) and the United Nations International Children’s Emergency Fund (UNICEF) recommend to initiate breastfeeding within the first hour after birth and to perform exclusive breastfeeding for the first 6 months [24, 25]. According to a study by Chung et al. the breastfeeding rates of South Korea have been increasing remarkably for the past 10 years [26]. Despite the increase in incidence, there has been a decrease in duration of breastfeeding, and there has been a continuous decrease in the 6-month exclusive breast feeding rate after the year 2003. The 6-month exclusive breastfeeding rate of South Korea is highest among Asian–Pacific countries but still lower than the US [26]. The results of our study show that breastfeeding affects various learning skills, even to the age of 8–11 years. These findings along with other studies reporting similar results emphasize the importance of encouraging further public health efforts to promote and support breastfeeding [3]. The development of effective guidelines for promoting breastfeeding and also education programs about breastfeeding for both health-care professionals and women undergoing childbirth could help increase the rate of breastfeeding and duration in South Korea [27].

This study has some noteworthy limitations. First, information pertaining to breastfeeding was assessed retrospectively when children were between the ages of 8 and 11, and responders may have been susceptible to recall bias. Due to the cross-sectional nature of this study, a causal relationship between breastfeeding and learning skills can’t be confirmed. Further studies using a prospective design can help minimize biases and establish a causal relationship. Furthermore, the group of breastfed children included those who were exclusively breastfed as well as those who were fed both by breast and by formula. Also, the variables related to SES (parental education, area of residence and income) did not reflect the SES at birth but rather the current SES. Further studies should collect data on the SES of participants at the time that breastfeeding was conducted. The LDES was administered by parents, and there were no teacher-rated or objective measures that could assess the child’s learning abilities. In addition, this study was an observational study and, although we adjusted for various gestational and socioeconomic factors, other subtle differences may have confounded the effects, including maternal behavior, interaction between mother and child and the degree of mother–child attachment. These factors are impossible to control for in observational studies. Previous studies have shown that the duration of breastfeeding has an impact on the effects of breastfeeding on cognitive ability, but information regarding the duration of breastfeeding was not available in our dataset. Onset of breastfeeding is also important, as the WHO recommends initiating breastfeeding in the first hour after birth, but no information on this matter was available.

## Conclusions

This is the first study to report that breastfeeding is associated with enhanced learning abilities in childhood-years, even after adjusting for maternal IQ. Future prospective studies that investigate home environments and the duration of breastfeeding are warranted to confirm the findings of this study.

## Abbreviations

IQ: intelligence quotient
SES: socioeconomic status
LDES: Learning Disability Evaluation Scale
LQ: learning quotient
DHA: docosahexaenoic acid
AA: arachidonic acid
